# The therapeutic potential of exosomes in immunotherapy

**DOI:** 10.3389/fimmu.2024.1424081

**Published:** 2024-07-08

**Authors:** Guangyao Li, Shuyi Zhang, Yitan Zou, Hongru Ai, Xinya Zheng, Kewen Qian, Changhai Lei, Wenyan Fu

**Affiliations:** ^1^ Department of Biophysics, College of Basic Medical Sciences, Naval Medical University (Second Military Medical University), Shanghai, China; ^2^ Department of Biomedical Engineering, College of Basic Medical Sciences, Naval Medical University (Second Military Medical University), Shanghai, China; ^3^ Department of Respiratory and Critical Care Medicine, Shanghai Changhai Hospital, Second Military Medical University, Shanghai, China; ^4^ Department of Assisted Reproduction, Shanghai Ninth People’s Hospital, Shanghai Jiao Tong University School of Medicine, Shanghai, China

**Keywords:** exosome (EXO), cancer, immune disease, COVID - 19, nanodrug

## Abstract

Exosomes are found in various tissues of the body and carry abundant contents including nucleic acids, proteins, and metabolites, which continuously flow between cells of various tissues and mediate important intercellular communication. In addition, exosomes from different cellular sources possess different physiopathological immunomodulatory effects, which are closely related to the immune regeneration of normal or abnormal organs and tissues. Here, we focus on the mechanistic interactions between exosomes and the human immune system, introduce the immuno-regenerative therapeutic potential of exosomes in common clinical immune-related diseases, such as infectious diseases, autoimmune diseases, and tumors, and reveal the safety and efficacy of exosomes as a novel cell-free immune regenerative therapy.

## 1 Introduction

**Figure 1:**
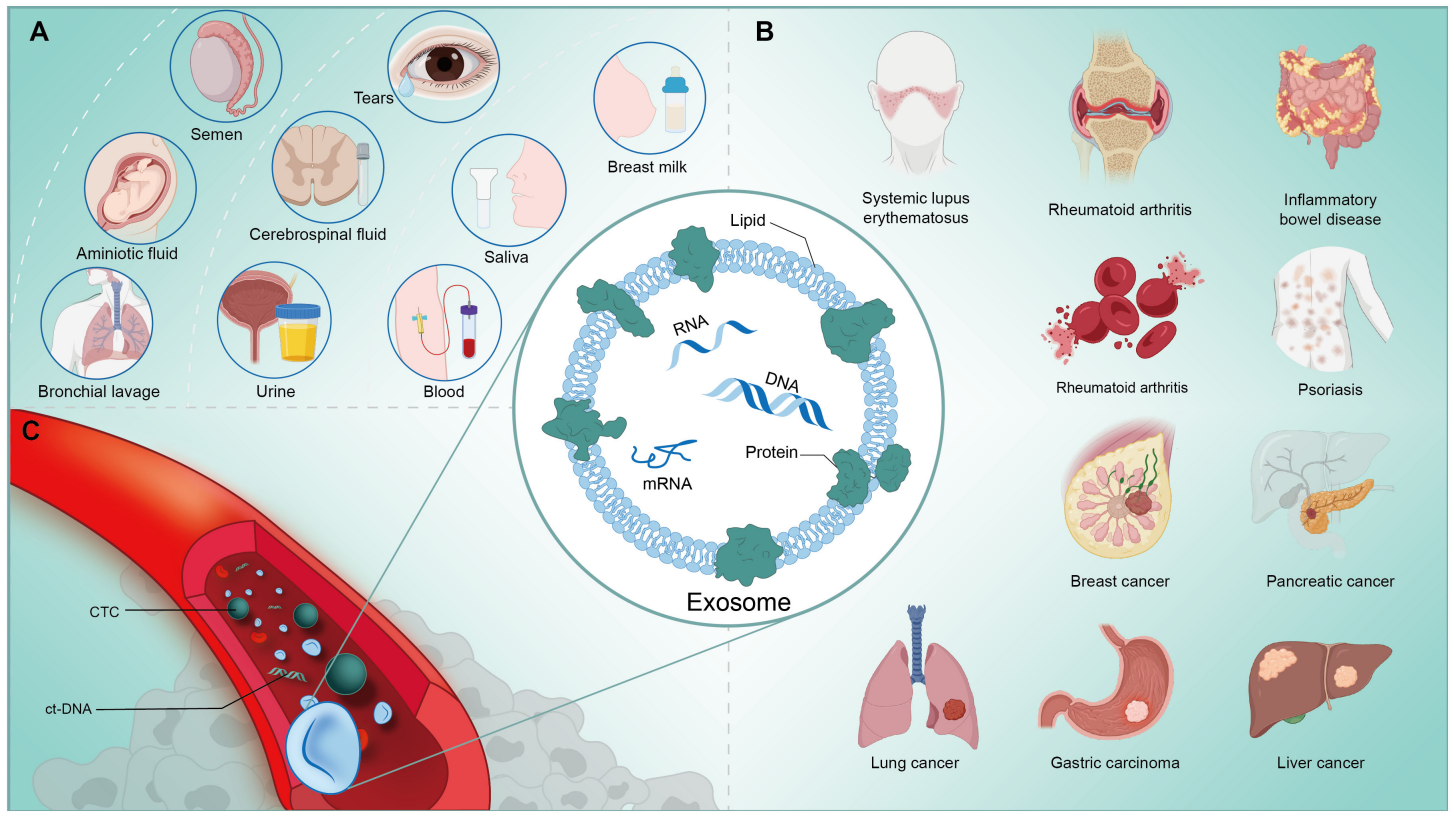
**(A)** Exosomes are widely present in the human body and can be extracted from a variety of body fluids, such as: tears, saliva, milk, blood, urine, etc. **(B)** Exosomes are closely associated with the physiological and pathological activities of the human body, and are involved in the process of immunoregulation in a variety of diseases, like cancers and autoimmune disorders. **(C)** Nucleic acids, proteins, metabolites and other components contained in exosomes extracted from human blood circulation have great potential to assist in the clinical diagnosis, process analysis and prognosis prediction of tumors and other diseases.

Extracellular vesicle (EV) is a general term for various types of vesicles secreted by all cells through membrane movement and contain various bioactive substances, including metabolic molecules such as proteins and lipids, and nucleic acids such as DNA and RNA (miRNA, circRNA, lncRNA) that contain genomic information. Specifically, the term exosome refers specifically to an endomembrane-derived intraluminal vesicle (ILV) that forms during the maturation of the multivesicular endosomal system (MEV) by inward fusion (1). These small-diameter vesicles have phospholipid bilayers with the same topological structure with appearance of elliptical or spherical, and will express molecules including CD63, CD81, CD9, Tspan8, compatibility complex (MHCIorII) and heat shock protein family (HSC70,HSP90), which are used as characteristic markers for identifying exosomes (2). According to the data of ExoCarta, as of Jan. 1, 2024, many exosome researches have been discovered and uploaded, including 9769 kinds of proteins, 3408 kinds of mRNAs, 2838 kinds of miRNAs and 1116 kinds of lipid molecules (3). Exosomes are not just a means for cells to renew themselves, but as an additional communication mechanism within and between cells, which participates in a large number of physiological and pathological changes closely related to clinical processes (4). Exosomes can persist in various body fluids besides peripheral blood, including but not limited to saliva, ascites, urine, cerebrospinal fluid, breast milk, etc. (**Figure 1A**) Therefore, as a cell replacement therapy, vesicles and its engineering derivatives have achieved breakthrough development in recent years, depending on its unique natural advantages, and have been applied to the diagnosis and treatment of clinical diseases, including cancer (5–7), neurological diseases (8–10), cardiovascular diseases (11) and Inflammatory disease (12). (**Figure 1B**) It is worth noting that engineered vesicle therapy regimens also seem to be making promising progress in the immune field.

## 2 Infectious disease

Immunotherapy for infectious diseases has become the focus again in recent years: the prevention and treatment of infectious disease like COVID-19. Over the past four years, the novel severe respiratory syndrome coronavirus type 2(SARS-CoV-2), which was born in 2019, has attracted widespread global public attention as a public health issue. Since the epidemic of the BA.5 variant in 2022, the coronavirus outbreak appears to have enjoyed a calmer period. However, data from a variety of sources suggest that we have entered a new wave of covid (13). Excitingly, the alternative strategy of another soluble receptor decoy, which possesses the advantage of ultra-breadth in maintaining neutralization ability against multiple variants of viruses, is expected to be a key strategy for solving the zoonotic coronavirus pandemic challenges in the future (14). There have been some well-established studies on the antiviral applications of engineered ACE2 decoy receptors, and their application in combination with EVs opens up new ideas for achieving superior multifunctional antiviral therapeutic effects. EVs carrying expressed ACE2 protein were extracted and characterized from a human lung epithelial cell line by Cocozza et al. Through *in vitro* antiviral assays, it was demonstrated that overexpression of full-length ACE2-EV and ACE2-TMPRSS2(Transmembrane protease, serine 2)-EV possessed significant viral inhibitory effects, equivalent to 500-1500-fold soluble recombinant ACE2 levels (15). ACE2 and palmitoyl secretion interact with each other through a strict mechanism; in other words, the presence of palmitoylation directly affects the strength of membrane-anchored ACE2 expression. Meanwhile, EVs containing ACE2 isolated from human plasma or cells have been shown to have a prophylactic capacity against severe acute SARS-CoV-2 that has been assessed to be 60- to 80-fold greater than that of vesiculation-free recombinant ACE2 (16). Moreover, the easily modifiable properties of EVs in terms of structure and function provide great potential for therapeutic performance enhancement, such as affinity and neutralization, of nano-neutralization devices carrying ACE2. Xie and his team used engineering to palmitoylate EVs carrying ACE2 to achieve a breakthrough in affinity for the S protein, as well as a broader neutralization of the virus in the host (17). This novel nano-delivery platform with palmitoylation as the core technology endows natural soluble ACE2 with superior half-life and *in vivo* stability (18). Kim et al. successfully constructed an engineered extracellular vesicular variant of sACE2 (sACE2.v1) using the CD9ΔTM4 scaffold and demonstrated its ability to cope with the infectious challenge of the WT, D614G, beta- and delta-variant strains in mice, exerting a significant protective effect. CD9ΔTM4 is a truncated form of CD9, serving as a scaffold whose C-terminus forms a splice with SACE2, and overexpression of the scaffold enables overpacking and functionalization of the fusion protein (19). Interestingly, in addition to the good progress in antiviral studies of soluble ACE2 with EVs, this research paradigm seems to be equally valid when applied to the structural domains of the viruses themselves that bind to ACE2, considering the binding of the S proteins to the host’s natural ACE2 receptor as a key prerequisite for viral invasion (20). In cellular experiments, EVs carrying viral spiny proteins were found to be significantly negatively correlated in a dose-dependent manner with antibody-mediated virus neutralization (21). Fu and colleagues successfully doped the receptor-binding domains (RBD) in the spike glycoprotein of SARS-CoV-2 onto extracellular vesicle membranes, and these RBD-labeled EVs were highly specifically targeted to ACE2-enriched tissues of the heart, lungs, and kidneys which were major organs infested by viruses and resulted in significant reductions of viral loads (22). In the field of vaccine development, a novel new crown candidate vaccine based on bacterial outer membrane vesicles (OMV), which is also coupled to the RBD of the viral spike, was shown to be protective in an intranasal inoculation model in mice, and retained better cross-neutralizing activity against WT and Delta for at least 35 days (23). In addition, lung-derived EV mounted messenger ribonucleic acid encoding viral spiking proteins has also been developed as a good room-temperature-stable vaccine product, also delivered by inhalation, which triggers an even better antibody secretion response (24). These findings demonstrate the versatility of the EVs delivery system in therapeutic form, somewhat circumventing the limitations of lung barrier utilization.

The therapeutic potential of EVs is not limited to their use as drug nanocarriers, and their physiological and pathological properties dictate that they play an important role in the process of tissue injury and inflammation, which is evidence for the source of their therapeutic potential in the treatment of SARS-CoV-2: to fight against COVID-19 by mitigating the pathological process of damage to lungs as the main infectious organ. Therefore, given the existence of natural protection of EVs for soluble ACE2 and the favorable properties of its derived therapeutic strategies, such as cell-free, immunologically safe, easily engineered, as well as multi-targeted therapeutic potential conferred by its lipophilicity and high clinical translational value (25, 26), the strategic model of doping ACE2 decoy receptors to antagonize COVID-19 will be of great value to explore.

## 3 Autoimmune disease

Mesenchymal stem cells (MSCs) are found in a variety of tissues in the human body and serve as multifunctional stem cells with renewal capacity and differentiation potential (27). A number of studies have shown that the immunomodulatory abilities of MSCs, including inhibition of inflammation progression, promotion of tissue repair, and resistance to pathogenic infections, etc., on immune effector cells as NK cells, B lymphocytes and T lymphocytes, are by means of the paracrine pathway, in which the secretion and communication of exosomes are the key parts (28–30). The treatment of autoimmune diseases has been controversial, and there are no recognized effective technological protocols. However, based on the positive modulatory effects of MSCs-derived exosomes on immunoreactive substances, several studies suggest that this novel cell-free therapy may be a powerful strategy against autoimmune diseases. Multiple sclerosis is the most common dysfunctional autoimmune disease with inflammatory involvement of the central nervous system (31). Microglia play a major immune role in the CNS, on the one hand their neuro-destructive role, represented by the M1 phenotype, and their anti-infective function against invading pathogens. At the same time, the M2 phenotype is responsible for inhibiting excessive pro-inflammatory responses to protect neurons from damage (32). In a study by Zhang et al, increasing the ratio of microglia M2 and M1 phenotypes by MSCs-derived exosomes adjustment was able to suppress neuroinflammatory progression in an animal model of experimental autoimmune encephalomyelitis (EAE). This combined direct and indirect immunomodulatory model significantly ameliorated neurodegenerative progression and optimized cognitive function compared to controls (33). For the powerful therapeutic role of MSCs-derived exosomes in EAE models, Riazifar’s team found that IFNγ-Exo was able to reduce the levels of multiple pro-inflammatory mediators *in vitro*, including Th1, IL-6, IL-22, and others. Meanwhile, in-depth characterization of exosomes, whose contents contain anti-inflammatory RNAs and a variety of protective neurological anti-inflammatory proteins, provides a molecular evidence base for their therapeutic mechanism (34). In addition, MSC-derived exosomes also have a new and safe prospect in the clinical treatment of Systemic Lupus Erythematosus (SLE). SLE, as a typical chronic autoimmune disease, has a complex and unknown pathogenesis and involved damaged organs, and nephritis is the main cause of disease and death of SLE patients at present (35). Therefore, the treatment of nephritis in patients with SLE is a key aspect of clinical suppression of disease progression. A growing body of research suggests that MSCs are a promising intervention strategy in the immunotherapy of lupus-like diseases. Transplantation of bone marrow-derived MSCs in animal models of lupus significantly reduced the abnormal levels of serum anti-double-stranded DNA antibodies and renal metabolites, which in turn induced potent positive immunomodulatory effects to alleviate the progression of lupus nephritis (36). A clinical trial in patients with drug-resistant systemic lupus erythematosus used a therapeutic strategy of allogeneic mesenchymal stem cell transplantation and followed 87 enrolled patients for an average duration of 27 months. The results showed significant remission of disease activity and clinical symptoms and improved organ function, reflected in an overall survival rate of up to 94%, confirming the excellent long-term safety and efficacy of this promising cell therapy (37). The above evidence suggests a promising application of MSCs in the clinical management of SLE patients, but there are still great challenges in terms of cost and regulation for their further dissemination and application. Interestingly, MSC-derived exosomes are the mainstay of the immunomodulatory effects of this therapy. Therefore, it is not difficult to arrive at the view that MSCs-derived exosomes, a novel cell-free therapy, is expected to break all the limitations in the future for the promotion and application of stem cell therapies in human SLE.

As mentioned previously, MSCs-derived exosomes exert immunomodulatory effects through communication interactions with immune cells, further influencing the expression levels of immunoreactive molecules, and this multifunctional modulatory ability to act simultaneously on both the natural and adaptive immune systems may be able to partially explain their strong therapeutic potential. Macrophages occupy an important position in the innate immune system and can be categorized into M1 pro-inflammatory and M2 anti-inflammatory phenotypes. Recent studies have shown that the anti-inflammatory effects of MSCs-derived exosomes are closely related to macrophage polarization, as evidenced by inhibition of M1 activation and induction of M2 immunosuppressive phenotype (38). Further, reduction of *in vivo* levels of immunoreactive substances such as IL22 and Th17, along with up-regulation of IL10, ultimately led to inflammatory suppression in macrophages (39, 40). In addition, MSCs-derived exosomes all possess the ability to inhibit the activation and proliferation processes of natural immune cells, such as NK and DC, thus exerting immunosuppressive effects and attenuating toxic inflammatory responses (41, 42). On the other hand, as the main immune cells of the adaptive immune system, both B cells and T cells were regulated by MSCs-derived exosomes, which in turn affected the functions of humoral and cellular immunity. Antibody secretion, activation, and proliferation of B cells were inhibited by MSCs, and interestingly, this regulatory effect could be perfectly inherited by MSCs-derived exosomes (43). At the same time, the secretion level of regulatory B cells, an important functional subpopulation, was significantly upregulated by exosomes, resulting in the release of large amounts of anti-inflammatory factors such as IL-10 (44). T cells are important participating effector immune cells in autoimmune diseases. Exosomes alleviate the progression of most autoimmune diseases by inhibiting T cell activation and proliferation. However, its role in regulating the proportion of helper T cells of different phenotypes is also crucial. The dynamic balance between Th1 and Th2 is closely related to the activation or suppression of inflammation. In addition, the increase in the proportion of Tregs and the induction of apoptosis in activated T cells also make an important contribution to disease prevention and delay in progression (45, 46).

## 4 Tumor

Cancer is a major threat to human life worldwide and has become a crucial challenge that hinders progress in average life expectancy and the maintenance of health (47). One of the main factors that make it difficult to control the number of cancer cases is the lack of potent screening tools for prevention (48). Precision medicine is advancing clinical research based on new biomarkers, with an emphasis on individualized treatment plans for patients (49). It is noted that the importance of intercellular communication of exosomes is not only reflected in physiological regulation, but also in strongly affecting the pathological progress of cells, tissues, organs, and even the biological behavior of tumors, including but not limited to cancer’s origin, metastasis and outcome (50) (**Figure 1C**). We have previously presented a detailed review of exosomes in immunotherapy of tumors, and we will not repeat it here, but mainly focus on the immunodiagnosis of exosomes in tumors (51).

As mentioned earlier, exosome contents are extremely rich, containing a variety of nucleic acids, proteins, metabolites, and other substances that harbor a wealth of genetic information and homeostatic changes. These complex substances and information are valuable tools for overcoming the difficulties of heterogeneous tumor diagnostic barriers. Firstly, exosomes can be potential biomarkers in cancer diagnosis and surveillance for high-risk metastasis. It has been well recognized that the detection of circulating miRNAs transported from cells can more precisely reflect the real-time dynamics of cancer cells for clinical diagnosis. Zhou et al. used Exiqon panel to analyze the miRNA expression profiles of plasma from lung adenocarcinoma(LA) patients and used reverse transcription-polymerase chain reaction (qRT-PCR) for further screening and validation, and successfully identified six miRNA groups that can distinguish LA patients from healthy control individuals by their dysregulation, providing a potential biomarker option for LA detection in Asian populations (52). The diagnostic assistance of extracellular vesicles in lung cancer is being validated in parallel in clinical trials (NCT04529915, NCT03830619). In addition to microRNA, long-stranded RNA in plasma is also a potential biomarker for predicting early cancer. The panel of serum exosomal 1 lncRNA, and 2 mRNA (BCAR4 and MAGEA3,KRTAP5-4) was shown to have unique predictive power in CRC (53). It has been reported that exosomal lnc PTENP1 in tissues and plasma of bladder cancer patients exhibits a strong correlation with tumor pathological features such as tumor morphology, size and weight, and maintains a significant negative correlation with clinical grade of the disease,(P < 0.05). Mechanistically, healthy cells secrete the exosome PTENPI to establish a pathological link with bladder cancer cells and protect PTEN by crosstalk with miR-17, and eventually the tumor malignant invasive behavior is oppressed. Therefore, its ability to frequently silence tumor cell lines allows it to be considered as a tumor suppressor (54). Secondly, different exosomal-biomakers can reflect the real-time treatment feedbacks and drug resistance detection of tumor. The complex dynamics of the tumor microenvironment is determined by the highly heterogeneous and mutagenic nature of cancer cells, whereas exosome biopsies are able to break through spatial and temporal limitations to systematically track dynamic changes in the heterogeneity of almost all cancers and influence the epigenetic characteristics and pathological properties of tumor cells through different regulatory mechanisms during the different clinical stages of conventional treatment of cancer patients (55). Circulating exosomal contents are stripped from primary tissue. Thus, it can serve as an easily accessible biomarker to assess patients’ clinical response to surgery, drugs and radiation therapy (56). Radioresistance is an important obstacle preventing NSCLC patients from achieving full therapeutic benefit. Extracellular miR-1246 was found to significantly inhibit the proliferation of lung cancer lineage tumor cells, while the radiosensitivity of tumor cells in miR-1246 knockdown models was enhanced, demonstrating the potential of *in vivo* miRNAs to regulate the sensitivity of lung cancer cells to radiation therapy (57). In addition, Alba et al. revealed a correlation between exosomal miRNA expression levels and breast cancer progression. The results confirmed significant differences in miR-21 expression levels between patients with localized and distant metastases of breast cancer(P=0.027), which corroborates the speculation that exosomal miRNAs can be used to predict risk stratification associated with cancer recurrence and metastasis (58). Since exosomes are highly consistent with cancer cells in terms of genetic heterogeneity, they can present comprehensive and macroscopic information as a whole, and also enable precise classification and screening of cell populations with different epigenetic characteristics at the microscopic level. The above results indicate that it is practical to reflect the health status of the organism by detecting the biological information contained in exosomes. In recent years, an increasing number of reports have shown that exosomes hold great promise in the field of clinical diagnosis.

## 5 Final discussion and perspective

However, there are a number of fundamental and translational issues that need to be considered and addressed before EV therapies can be applied to humans on a large scale. Firstly, in the optimization and screening of EV-sACE2 engineered samples, the relationship between ACE2 loading and inhibitory potency remains controversial in different research reports. In contrast to the generally accepted result that increasing ACE2 loading significantly improves neutralization, improving antiviral performance by increasing ACE2 loading in a certain population of vesicles of a certain size may not be satisfactorily rewarded. For example, in Gunnels’ study, there was no significant difference in potency of specific ACE2 between different types of loaded vesicles, which contradicts the conclusion that ACE2 loading is positively correlated with potency as reported by Xie et al. (17, 59). Therefore, the means of EV purification and size type should be included in platform design considerations to exclude their possible unintended effects on performance. The design of exosome targeting optimization is challenging and some rational targeting strategies have potential. For example, the chemical structure of exosomes can be optimized to increase their permeability and retention and reduce clearance. In addition, investigating more bioactive nanocoated materials to improve exosome targeting and utilization are important design factors (60). In addition, there is no standardized consensus among the many different studies and companies on protocols for isolation and characterization of EVs, which makes it difficult to ultimately reach a consistent concentration and pattern of EVs even when they are derived from the same tissue or cell. For instance, even the most classical differential ultracentrifugation scheme suffers from low productivity and operator randomness for EV separation applications (61). Therefore, a universal economic Good Manufacturing Practices (GMP) guideline is exactly what is urgently needed by research and industry. Finally, given the heterogeneity that exists in different diseases EV therapies need to determine the optimal dose and route of administration to match the specific disease. Specific clinical guidance on exosomes, a novel cell-free therapy, is not yet available in Asia, Europe or the United States, but it is expected that their origin and function will determine the type of regulatory framework to which they are subject (62). In the validation of lung injury models, both the intravenous and inhalation routes possessed better therapeutic efficacy, but the latter may have better organ local targeting in order to seek to achieve precise high-concentration delivery to localized lesions.

Overall, novel EV-based alternative therapies hold good promise in dealing with treatment and diagnosis of clinical immune diseases like viral infections (COVID), autoimmune diseases and cancers because of their excellent maneuverability and safe action properties that penetrate deep into pathophysiological mechanisms. It is foreseeable that EV therapeutics will be a powerful immune weapon for human beings to cope with clinical diseases, after determining the optimal safe pathway for preparation, characterization and application.

## Data availability statement

The original contributions presented in the study are included in the article/supplementary material. Further inquiries can be directed to the corresponding authors.

## Author contributions

GL: Writing – original draft, Writing – review & editing. SZ: Resources, Writing – review & editing. YZ: Investigation, Writing – review & editing. HA: Data curation, Writing – review & editing. XZ: Investigation, Writing – review & editing. KQ: Formal analysis, Writing – review & editing. CL: Writing – review & editing. WF: Writing – review & editing.
